# Towards estimates of future rainfall erosivity in Europe based on REDES and WorldClim datasets

**DOI:** 10.1016/j.jhydrol.2017.03.006

**Published:** 2017-05

**Authors:** Panos Panagos, Cristiano Ballabio, Katrin Meusburger, Jonathan Spinoni, Christine Alewell, Pasquale Borrelli

**Affiliations:** a European Commission, Joint Research Centre, Directorate for Sustainable Resources, Via E. Fermi 2749, I-21027 Ispra (VA), Italy; b Environmental Geosciences, University of Basel, Switzerland

**Keywords:** R-factor, Climate change, Rainfall intensification, Storminess, RCP4.5, Erosion scenario

## Abstract

The policy requests to develop trends in soil erosion changes can be responded developing modelling scenarios of the two most dynamic factors in soil erosion, i.e. rainfall erosivity and land cover change. The recently developed Rainfall Erosivity Database at European Scale (REDES) and a statistical approach used to spatially interpolate rainfall erosivity data have the potential to become useful knowledge to predict future rainfall erosivity based on climate scenarios. The use of a thorough statistical modelling approach (Gaussian Process Regression), with the selection of the most appropriate covariates (monthly precipitation, temperature datasets and bioclimatic layers), allowed to predict the rainfall erosivity based on climate change scenarios. The mean rainfall erosivity for the European Union and Switzerland is projected to be 857 MJ mm ha^−1^ h^−1^ yr^−1^ till 2050 showing a relative increase of 18% compared to baseline data (2010). The changes are heterogeneous in the European continent depending on the future projections of most erosive months (hot period: April–September). The output results report a pan-European projection of future rainfall erosivity taking into account the uncertainties of the climatic models.

## Highlights

• Rainfall erosivity in Europe & Switzerland is estimated to increase by 18% in 2050.
• Rainfall erosivity will increase in 81% of the study area and decrease in the rest.
• R-factor projections include the uncertainty of climatic models.
• Highest R-factor increase is projected in Northern & Central Europe.
• Erosivity is a driver for soil erosion, floods, natural hazards & land use change.

## Graphical abstract

**Figure:**
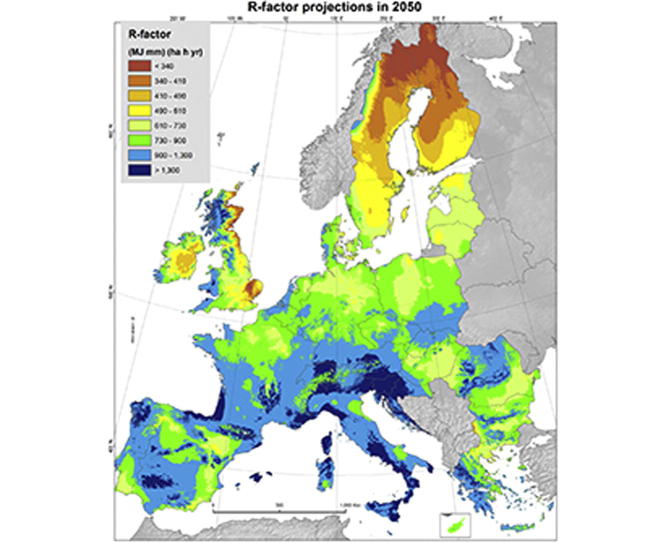

## 1 Introduction

Soil erosion is one of the main European environmental threats, particularly in Southern Europe (Panagos et al., 2015a). Its prevention and mitigation is a key ecosystem service to monitor and access spatially and temporally (Guerra et al., 2016). Accelerated soil erosion may lead to a decrease of ecosystem stability, land productivity, land degradation in general and a loss of income for farmers (Salvati and Carlucci, 2013). Soil erosion and more generally land degradation is driven by unsustainable land management due to increasing human pressure enhanced by climate change (Helldén and Tottrup, 2008). The extent, frequency and magnitude of soil erosion in Europe is expected to increase due to a general increase of extreme rain fall events caused by climate change (Pruski and Nearing, 2002, Deelstra et al., 2011).

The prediction of soil erosion changes in the future are mainly dependent on modeling future rainfall erosivity, land use changes and impacts of policies on soil loss. The most commonly used erosion models are the the various types of the Universal Soil Loss Equation (USLE) originally developed by Wischmeier and Smith (1978). In the proposed algorithms, soil loss by water erosion is proportional to rainfall erosivity (R-factor), which is one of five input factors. While rainfall erosivity accounts for the effect of rainfall in soil erosion, the soil erodibility (K-factor) incorporates the soil properties defining the susceptibility of a soil to erode, the cover management (C-factor) takes into account the land use and management in agricultural lands, the slope length and steepness (LS-factor) accounts for the topography and finally the support practices (P-factor) considers the effect of conservation measures. A modified version of the USLE, the Revised Universal Soil Loss Equation (RUSLE), was originally suggested by Renard et al. (1997), and has been recently applied in Europe (RUSLE2015) for the estimation of soil loss by water at 100-m resolution (Panagos et al., 2015a). Among other improvements compared to past Pan-European soil erosion assessments, RUSLE2015 incorporates the option of running climate change, land use change and policy scenarios.

Rainfall erosivity is a multi-annual average index that measures rainfall kinetic energy and intensity describing the effect of rainfall on sheet and rill erosion (Wischmeier and Smith, 1978). The rainfall erosivity of a given storm in RUSLE (referred to as R-factor) is equal to the product of the total storm energy with its maximum 30-minutes rainfall intensity. As high temporal resolution rainfall data are commonly not available, many studies estimated rainfall erosivity using approximation equations based on monthly or daily rainfall data (Bonilla and Vidal, 2011, Diodato and Bellocchi, 2010). Only recently, R-factors were directly estimated from high temporal resolution data at national/regional scale in Europe such as the study in Slovenia (Petan et al., 2010), Switzerland (Meusburger et al., 2012), Ebro catchment in Spain (Angulo-Martinez et al., 2009), Czech Republic (Janeček et al., 2013), Greece (Panagos et al., 2016a) and Italy (Borrelli et al., 2016).

The occurring and projected climate change is likely to affect soil erosion due to intensification of rain, change of precipitation amounts, change of moisture and vegetation cover change (St.Clair and Lynch, 2010). The most important impact of climate change on soil erosion is expected due to an increase of rainfall intensity, in particular the increase of extreme rainfall events both at global (e.g., Sillmann et al., 2013) and continental scale (for Europe see, e.g., Frei et al., 2006, Westra et al., 2014). There are few studies which have addressed the risk of increasing rainfall erosivity based on past trends (Verstraeten et al., 2006 in Ukkel, Belgium; Fiener et al., 2013 in western Germany; Hanel et al., 2015 in Czech Republic). At national scale, future trends in rainfall erosivity were addressed in the USA (Nearing, 2001, Biasutti and Seager, 2015), China (Zhang et al., 2010) and Japan (Shiono et al., 2013). The studies in USA used mean annual precipitation combined with Fournier coefficient (Arnoldus, 1980) while the ones in China and Japan have downscaled the monthly precipitation spatially and temporally. The improved understanding of General Circulation Models (GCM) and the increased data availability contributed to their wider use and allow for their integration in ecological-related disciplines (e.g. soil, water). For instance, Nearing (2001) has applied the HadCM3 climate change scenario (Gordon et al., 2000) and estimated increases in rainfall erosivity between 16% and 58% in the USA.

The objective of this study is to estimate the expected change in rainfall erosivity and its impact on soil erosion in Europe during the first half of the 21st century based on the updated IPCC climate change scenarios (IPCC, 2013). This study focuses on the R-factor changes without considering the impact of climate change on land/ vegetation cover. Compared to previous studies that used approximation equation based on annual (or monthly) precipitation, this study use as input the high-temporal-resolution Rainfall Erosivity Database at European Scale (REDES) (Panagos et al., 2015b) and climatic data derived from the WorldClim database, which is set of global climate grids with a spatial resolution of about 1 km^2^ (Hijmans et al., 2005).

## 2 Database and modelling approach for R-factor prediction

This chapter presents: a) a brief description of REDES and its latest updates; b) WorldClim datasets modelling future climatic conditions; c) the climate projections for 2050 in Europe with specific focus on rainfall, and d) the regression model applied for the R-factor future prediction.

### 2.1 Rainfall Erosivity Database at European Scale (REDES)

The first version of the Rainfall Erosivity Database at European Scale (REDES) (2014) included 1,541 rainfall stations within the European Union (EU) and Switzerland (Panagos et al., 2015b). In 2015, an update of REDES was performed with 134 new R-factor stations, which resulted in 1675 REDES stations. The spatial distribution and the density of rainfall stations in REDES is not homogeneous in all EU countries (Ballabio et al., 2017) due to availability (or not) of high temporal resolution rainfall data. Auerswald et al. (2015) addressed 5 comments on REDES dataset and Panagos et al. (2015c) replied to this. Both studies (Auerswald et al., 2015, Panagos et al., 2015c) agree that the use of a short time series or time series from different periods is generally a problem in all large-scale studies and requires improvement in the future.

The R-factor as a proxy for rainfall erosivity has been calculated in REDES by using high temporal resolution data (5-min, 10-min, 15-min, 30-min and 60-min) and applying the equations proposed by Brown and Foster (1987). The R-factor is the product of the kinetic energy of a rainfall event (E) and its maximum 30-minutes intensity (I_30_) (Brown and Foster, 1987).

### 2.2 WorldClim datasets: Baseline and projected

Global precipitation and temperature (both annually and monthly) at a high spatial resolution of 1 km^2^ are available from the WorldClim database (Hijmans et al., 2005). The data layers are generated through interpolation of average monthly climate data from weather stations on a 30 arc-second resolution grid (referred to as “1 km^2^” resolution) and include precipitation data from 47,554 locations and maximum/minimum temperature data from 14,835 locations all over the world. The density of stations in Europe is among the highest ones. Records for at least 10-years have been used to calculate the average monthly climatic grids, which represent the baseline climatic situation.

**Fig. 1:**
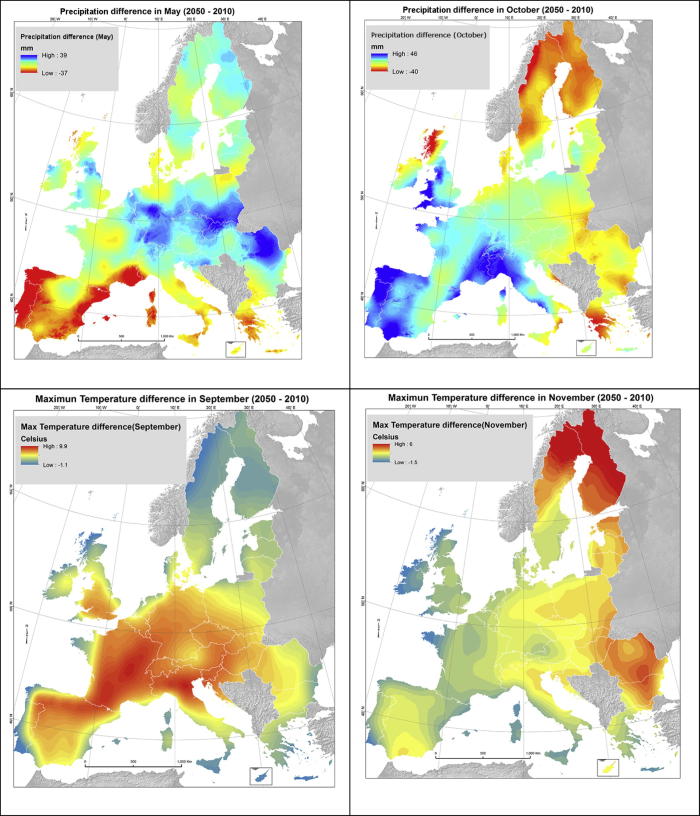
Examples of climate change predictions according to WorldClim datasets: differences between 2050 projections and baseline are shown for: a) the precipitation in May , b) precipitation in October , c) Maximum temperature in September, d) Maximum Temperature in November.

The future climate projection for 2050 are derived from General Circulation Models such as HadGEM2 (see next section) that was used in this study (Milton et al., 2011). The yielded projections are downscaled and calibrated (bias-corrected) using WorldClim as the historical (1950–2000) baseline (Hijmans et al., 2005). The datasets on future projections refer to the middle century (2041–2060) and the midpoint year 2050 will be used as reference in the following. Difference maps of WorldClim datasets (future projections compared to baseline ones) show the impact of climate change in precipitation and temperature (Fig. 1).

### 2.3 Climate projections in Europe

The Intergovernmental Panel on Climate Change (IPCC) recently published the 5th assessment report in 2013–14 (IPCC, 2013), describing the projections of climate change during the 21st century. Climate projections are model-driven descriptions of possible future climates under a given set of plausible scenarios of climate change (Weaver et al., 2013, Rummukainen, 2010).

General Circulation Models (GCMs), as well as the Regional Circulation Models (RCM) represent powerful tools to produce spatially explicit predictions on future climate changes based on a given scenario. More than 50 General Circulation Models are currently available for environmental studies. GCMs are numerical representations of climate systems based on physical, chemical and biological properties of oceans, land and ice surface (Harris et al., 2014). Among the 50 GCMs, we have selected the HadGEM2 climate model developed by Met Office Hadley Centre in United Kingdom (Martin et al., 2011, Jones et al., 2011). HadGEM2 represents the current state of the art and it is a valuable tool for predicting future climate and understanding the climate feedbacks within the earth system (Milton et al., 2011).

The climate change scenarios are called Representative Concentration Pathways (RCPs) and the 3 main used RCPs are RCP2.6, RCP4.5 and RCP8.5. Among these 3 prevailing climate change scenarios, we have selected the RCP4.5 which is the most widely used and which is neither conservative (RCP2.6) nor extreme (RCP8.5). The RCP4.5 scenario forecasts an increase in greenhouse gases that is expected to peak around 2040, afterwards a smooth decline until the end of the century is assumed.

The RCP4.5 scenario applied with the General Circulation Models HadGEM2 and calibrated with WorldClim baseline data projects a global mean surface temperature increase by 1.4 Celsius degrees (range 0.9–2.0) in the period 2046–2065 and by 1.8 Celsius degrees (range 1.1–2.6) in the period 2081–2100 compared to the reference period of 1986–2005 (IPCC, 2013). The projected mean global increase in extreme precipitation events by 10% and an increase of global precipitation amount by 5% by the end of 21st century is relevant for rainfall erosivity changes (Kharin et al., 2013).

### 2.4 Relating R-factor and WorldClim climatic data with Gaussian Process Regression

Since intensity, duration, frequency and amount of rainfall has large uncertainty in future predictions, and the General Circulation Models (GCMs) lack temporally high resolved data (<1 h) for a direct R-factor estimation, the application of statistics and stochastic approaches represent an alternative to predict the potential change in R-factor. Previous attempts to estimate changes in R-factor at catchment and national scale (Nearing, 2001, Zhang et al., 2010 Ito, 2007) have used relationships between rainfall erosivity and monthly or annual rainfall. However, those relationships do not consider the changes in rainfall intensity and the frequency of storm events (Shiono et al., 2013).

**Fig. 2:**
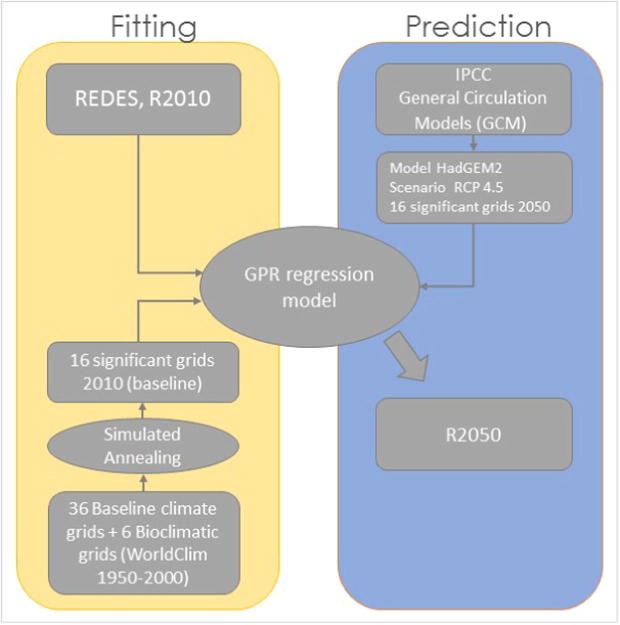
Procedure followed to project future (2050**)** rainfall erosivity for Europe.

Here we follow a different approach (Fig. 2), because we found in a previous study that rainfall erosivity (R-factor) is strongly correlated with precipitation dynamics (precipitation seasonality, monthly precipitation) in Europe (Panagos et al., 2015b). In this study, we chose a regression approach to derive the distribution of rainfall erosivity in 2050 (dependent variable) from a series of related but independent WorldClim climatic variables (covariates). This is done by fitting a regression model using baseline climatic conditions derived from the WorldClim dataset and the rainfall erosivity as calculated from field measurements.

The GPR regression model establishes a statistical relation between the R-factor point values (calculated from REDES) and WorldClim baseline climatic data acting as a set of spatially exhaustive covariates (Fitting part in Fig. 2). In a second step, this GPR regression model is applied to WorldClim future climatic data layers for the year 2050 (HadGem2, Scenario 4.5) in order to derive the future predictions of the rainfall erosivity (R2050) (Prediction part in Fig. 2)

The rationale behind this procedure is that rainfall intensities and as such rainfall erosivity are associated with given combinations of climatic conditions that occur in the present. It is assumed that in the future, similar combinations of climatic conditions are related in the same way to rainfall intensities and rainfall erosivity but will likely occur at different latitudes or in different periods of the year. Consequently, applying the regression model fitted on current climatic dataset allows to estimate future levels of rainfall erosivity when the same model is applied with covariates of projected future climatic data sets.

The Gaussian Process Regression (GPR) was used as regression method in this study. It is a regression technique generally suited for large scale applications where high dimensionality (number of degrees of freedom) of data used and non-existence of linear relationships between target variable and covariates (Vasudevan et al., 2009, Rasmussen and Williams, 2006) subsists. The GPR model was selected in this study for two reasons: a) better performance (in terms of R2, RMSE, Standard error) compared to other models and b) comparability of results with the existing rainfall erosivity in Europe where GPR was also applied (Panagos et al., 2015b). The details on how the regression model Gaussian Process Regression is applied for the rainfall erosivity prediction are described in the rainfall erosivity in Europe (Panagos et al., 2015b).

In this model application, the optimization of the GPR by feature selection was performed using a Simulated Annealing (SA) approach (Kirkpatrick et al., 1983). Simulated Annealing (SA) is an optimization technique processing arbitrary degrees of nonlinearities (and stochasticity) and guarantees to find the statistically optimal solution (Ingber, 1993). Further, SA allows finding the best set of covariates to be included in the GPR model by optimizing a chosen model metric; in this case the metric is cross-validation Root Mean Square Error (RMSE).

For the first fold, *n-1* of the data is used in the search while the remaining *n-(n-1*) is used to estimate the internal performance. The fitted model is then applied to all the data in order to obtain the external performance. This allows having two metrics, one used for fitting the model (internal performance) and the other used to express global model performance. SA also allows to estimate variable importance by ranking variable frequency candidate models through the optimization process and their influence on the final model. Finally, the GPR equation together with the projected changes of the same covariates will be used to estimate R-factor in 2050.

The GPR could potentially use 42 covariates from the WorldClim database. Among them, the 36 monthly layers represent the following 3 climatic variables (each one has 12 monthly layers):

- monthly total precipitation (mm)
- monthly average minimum temperature (degrees C * 10)
- monthly average maximum temperature (degrees C * 10)

Moreover, we have used bioclimatic variables which are derived from monthly temperature and precipitation values and generate biologically meaningful variables of WorldClim. Those bioclimatic variables represent annual trends, seasonality and extreme or limiting environmental factors. In the prediction of rainfall erosivity, we have used six bioclimatic variables a) the Mean diurnal range (Mean of monthly difference between maximum and minimum temperature), b) isothermality c) temperature seasonality (standard deviation * 100), d) precipitation seasonality (Coefficient of variation) e) precipitation of warmest quarter (period of 3 months; ¼ of the year) and f) precipitation of the coldest quarter. The six bioclimatic variables are pre-selected among the nineteen available ones as they are not collinear with the monthly precipitation and temperature values which have been already included in the model.

## 3 Results and discussion

### 3.1 Gaussian Process Regression fitting

**Fig. 3:**
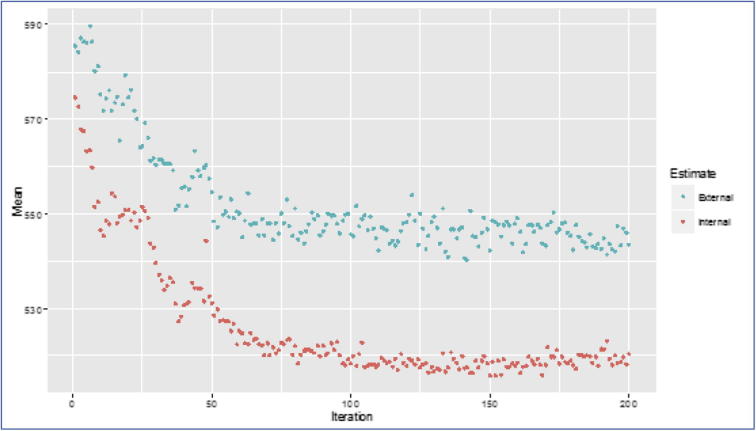
Optimization profiles of the SA. The vertical axis expresses the average RMSE result of internal and external cross-validation.

The Simulated Annealing (SA) procedure has been applied over the set of 42 proposed WorldClim covariates (see 2.4 Section) and a selected set of 16 covariates was used in the final best model (Table 1). The selection procedure converges at iteration 150 where the minimum RMSE of 515.78 is reached (Fig. 3) for external validation. The stability of the model output is supported by the plateau reached between the 100th and the 200th iteration (Fig. 3). This assures the good performance of the model in generalizing properties (such as future R-factor) and reducing the likelihood of runaway estimations in predicting future rainfall intensities (unless forecasted climatic variables with runaway values are provided as input to the model). The overall performance of the model is evidenced by an R^2^ of 0.635, while the relative error is 0.56 for the entire dataset (Fig. 3).

**Table 1:** Ranking of WorldClim variables according to the Simulated Annealing (SA) optimization. Variables are ranked according to their respective selection frequency.

Parameter	Covariate explanation	Selection frequency	Included in the model (Y)es/(N)o
Prec8	Average precipitation (mm) in August	80	Y
Prec4	Average precipitation (mm) in April	80	Y
Bio15	Precipitation Seasonality	80	Y
Tmin3	Average minimum temperature in March	70	Y
Prec9	Average precipitation (mm) in September	70	Y
Prec7	Average precipitation (mm) in July	70	Y
Prec6	Average precipitation (mm) in June	70	Y
Prec5	Average precipitation (mm) in May	70	Y
Bio3	Isothermality	70	Y
Bio18	Precipitation (mm) of Warmest Quarter	70	Y
Tmin6	Average minimum temperature in June	60	Y
Tmin2	Average minimum temperature in February	60	Y
Tmax8	Average maximum temperature in August	60	Y
Prec2	Average precipitation (mm) in February	60	Y
Prec11	Average precipitation (mm) in November	60	Y
Bio4	Temperature Seasonality	60	Y
Tmin9	Minimum temperature in September	60	N
Tmax6	Average maximum temperature in June	50	N
Tmax5	Average maximum temperature in May	50	N
Tmax2	Average maximum temperature in February	50	N
Tmax12	Average maximum temperature in December	50	N
Tmax10	Average maximum temperature in October	50	N
Prec10	Average precipitation (mm) in October	50	N
Prec1	Average precipitation (mm) in January	50	N

The best model includes 16 variables ranked as shown in Table 1. We observed that if more variables are included in the model, it is not implied that the performance will be improved.

Among the 16 variables for the application of the future prediction R-factor model at European scale, 8 monthly precipitation datasets are included. It is notable that precipitation of winter months (December, January), March and October are not included in the model while the warmer months during the vegetation period (April to September) are included. Regarding the temperature effect in the future predictions of rainfall erosivity at European scale, the GPR model included 3 monthly minimum average temperatures (February, March and June) and only one monthly maximum average temperature (August). The GPR model included also four out of six bioclimatic variables in R-factor predictions: a) isothermality (Mean Diurnal Range divided by Temperature Annual Range) b) temperature seasonality (standard deviation) c) precipitation seasonality (Coefficient of Variation) and d) precipitation (mm) of warmest quarter.

### 3.2 Rainfall erosivity in Europe in 2050

**Fig. 4:**
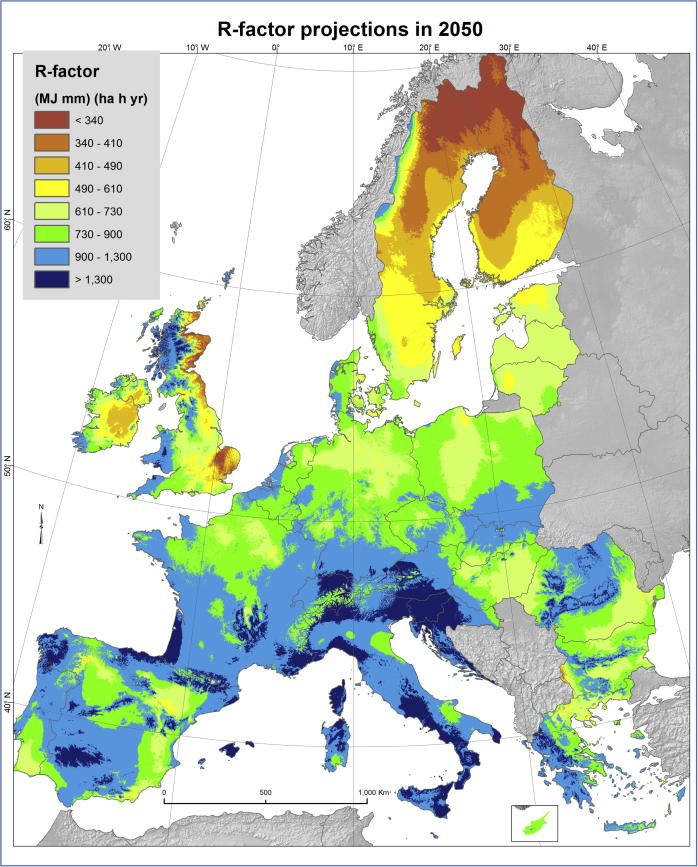
Rainfall erosivity projection for the year 2050 according to RCP 4.5 scenario driven by the HadGEM2 GCM model.

The projected rainfall erosivity based on REDES and WorldClim datasets according to RCP 4.5 climate change scenario driven by the HadGEM2 GCM model (Fig. 4) shows an increase of the R-factor in Northern and Central European countries. The projected mean R-factor for 2050 in the European Union and Switzerland is 857 MJ mm ha^−1^ h^−1^ yr^−1^ showing an increase of 18% compared to the current rainfall erosivity (Panagos et al., 2015b). The mean absolute error was estimated at 319 MJ mm ha^−1^ h^−1^ yr^−1^ and the relative error 0.56 with a model R^2^ of 0.64. A simulation with the RCP 2.6 climate change scenario showed a smoother increase of erosivity compared to baseline (16%) while the use of the most aggressive scenario RCP 8.5 showed a notable increase of erosivity (27%).

### 3.3 Estimated changes of rainfall erosivity in Europe (2050 compared to 2010)

**Fig. 5:**
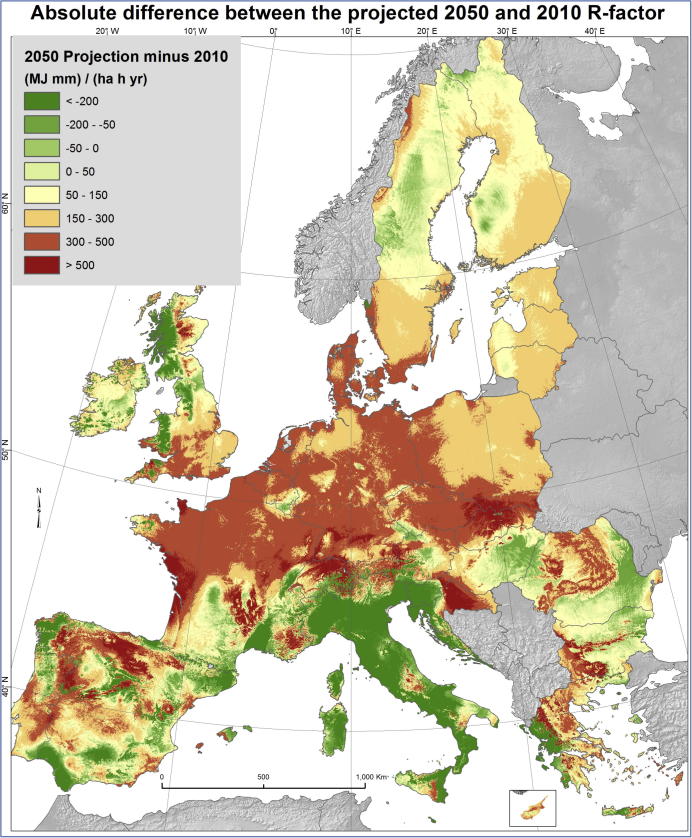
Absolute difference of R-factor between 2050 projections and 2010 data.

Besides the future projections of rainfall erosivity, it is important to highlight the change compared to baseline dataset of 2010 (Fig. 5). This comparison is feasible because the same GPR model is used but with different climatic input conditions (2010 versus 2050 climatic data). The absolute difference in R-factor between the 2050 projection and 2010 baseline allows to identify areas of strong erosivity decrease or increase (Fig. 5). Based on this assessment, 81% of the area in Europe (around 3.5 * 10^6^ Km^2^) is predicted to have an increased rainfall erosivity by 2050 and only for the remaining 19% rainfall erosivity is predicted to decrease (Fig. 5). In almost 25% of the study area the R-factor is increasing by at least 50% by the year 2050 compared to the baseline data (2010).

In large parts of Italy and Slovenia, Western Croatia (Adriatic sea), Scotland, eastern Spain, eastern Bulgaria, eastern Romania, Western Greece and North West Iberian Peninsula a pronounced decrease of the absolute rainfall erosivity is expected (Fig. 5). Most of those areas (Scotland, Italy, Slovenia, Western Greece, Croatia and North west Iberian Peninsula) have very high mean R-factor (>1,300 MJ mm ha^−1^ h^−1^ yr^−1^) in 2010 (Panagos et al., 2015b) and the projected decrease is more than 200 MJ mm ha^−1^ h^−1^ yr^−1^ till 2050 mainly due to less rainfall.

The potentially most problematic areas are probably the ones where an increase of more than 500 MJ mm ha^−1^ h^−1^ yr^−1^ is projected by 2050. The higher rainfall erosivity in these areas is caused by more intense rainstorms and/or by more frequent erosive events. The Swiss Alps, part of the French Atlantic coast, East Croatia and parts of Slovakia and southern Germany are expected to have such increase (rainfall intensity and/or frequency of erosive events), according to the most often applied RCP4.5-based scenarios (IPCC, 2013), including also the HadGEM2 GCM used in this study. In major parts of the North Europe (France, Belgium, Netherlands, Germany, Denmark and Czech Republic) a notable increase of rainfall events during summer period is going to increase erosivity by 300–500 MJ mm ha^−1^ h^−1^ yr^−1^. According to the ratio of current erosivity compared to future (till year 2050) R-factor, in those areas it is expected to double. In Baltic states and Poland this increase will be lighter but quite pronounced compared to the nowadays rates.

The highest mean relative increase (>50%) in rainfall erosivity by 2050 is projected for the Netherlands, Denmark, Czech Republic, Slovakia, Germany and Poland (Table 2). A decrease in mean rainfall erosivity is projected in Italy, Malta and Slovenia (>20%). In Spain and Greece, a slight increase of mean rainfall erosivity is projected while in Ireland the situation remains fairly stable.

**Table 2:** Mean R-factor values estimated for current climatic conditions (2010**)** and for the projected future scenario RCP4.5 (2050**)** per country.

Country		Mean R-factor (2010)	Mean projected R-factor (2050)	Change (%) 2010–2050
		MJ mm ha^−1^ h^−1^ yr^−1^		
AT	Austria	1,075.5	1,240.8	15.4%
BE	Belgium	601.5	881.9	46.6%
BG	Bulgaria	695.0	838.2	20.6%
CH	Switzerland	1,039.6	1,290.9	24.2%
CY	Cyprus	578.1	817.0	41.3%
CZ	Czech Republic	524.0	883.5	68.6%
DE	Germany	511.6	849.8	66.1%
DK	Denmark	433.5	772.3	78.2%
EE	Estonia	444.3	620.5	39.7%
ES	Spain	928.5	1,013.4	9.1%
FI	Finland	273.0	404.1	48.1%
FR	France	751.7	999.1	32.9%
GR	Greece	827.7	949.8	14.8%
HR	Croatia	1,276.2	1,297.6	1.7%
HU	Hungary	683.3	759.3	11.1%
IE	Ireland	648.6	654.6	0.9%
IT	Italy	1,642.0	1,249.5	−23.9%
LT	Lithuania	484.2	686.5	41.8%
LU	Luxembourg	674.5	945.2	40.1%
LV	Latvia	480.4	664.3	38.3%
MT	Malta	1,672.4	1,277.3	−23.6%
NL	Netherlands	473.3	841.1	77.7%
PL	Poland	537.1	814.4	51.6%
PT	Portugal	775.1	960.4	23.9%
RO	Romania	785.0	930.2	18.5%
SE	Sweden	378.1	494.6	30.8%
SI	Slovenia	2,302.0	1,780.2	−22.7%
SK	Slovakia	579.7	971.9	67.7%
UK	United Kingdom	746.6	780.0	4.5%

An analysis per main climatic zones in Europe (EEA, 2011) shows that the Boreal, Continental and Atlantic regions will be relatively more affected by increased rainfall erosivity by 2050 (Table 3). The Alpine climatic zone will show an increase of 13% of the R-factor and will be the area with highest mean rainfall erosivity (approximately 1056 MJ mm ha^−1^ h^−1^ yr^−1^ by the year 2050). The areas around the Adriatic Sea (Italian coast, Slovenia, Croatia and Western Greece) show a notable decrease of rainfall erosivity. The mean R-factor in the Mediterranean zone remains stable with different spatial patterns.

**Table 3:** R-factor projections estimated for current climatic conditions (2010) and for the projected future scenario RCP4.5 (2050) per Biogeographical region.

Climatic Zone	Proportion of the study area	Mean R-factor (2010)	Mean projected R-factor (2050)	Change (%)
	%	MJ mm ha^−1^ h^−1^ yr^−1^		
Alpine	9.2	932.3	1056.5	13.3%
Atlantic	17.7	678.2	863.2	27.3%
Black Sea	0.2	702.1	772.7	10.1%
Boreal	19.1	359.5	492.5	37.0%
Continental	29.7	695.7	911.2	31.0%
Mediterranean	20.4	1050.6	1048.5	−0.2%
Pannonian	2.9	660.1	754.5	14.3%
Steppic	0.8	729.8	686.6	−5.9%

### 3.4 Model uncertainty

**Fig. 6:**
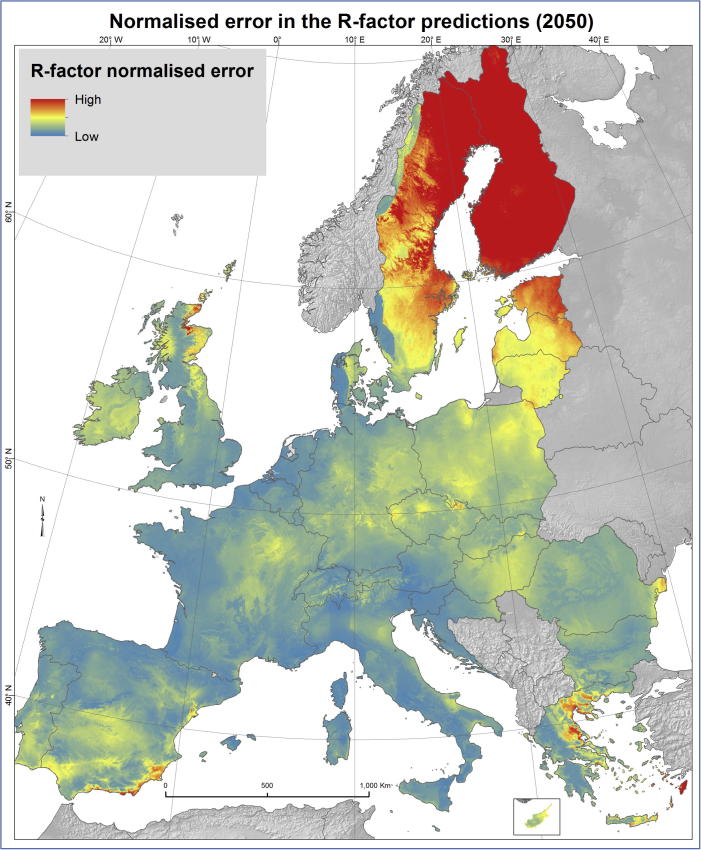
Normalised error in the R-factor prediction (2050).

The uncertainty of the predictions has been quantified by modelling the normalised error of the R-factor predictions (Fig. 6). The GPR model has the advantage to estimate both the prediction of the mean and the prediction of the mean variance. The standard error map expresses how much the estimated value of R-factor might vary. We expressed this variation as a proportion of the estimated R-factor value (Fig. 6). Likely, areas with a high error are those where the model has to make predictions on a combination of climatic factors that are not present in 2010, the baseline situation. Thus, areas where largest changes are predicted by the GCM are likely to have a high uncertainty. Indeed, the areas with higher uncertainty are the Scandinavian countries, Baltic States, Scotland and part of Greece and Spain. Medium uncertainty is noticed in Poland, parts of Germany, Czech Republic, Hungary, Central France and southern Iberian Peninsula. However, it should be noted that the high normalised error values in Scandinavia are due to the very low absolute estimated value of R-factor in that area and might thus not be of a high relevance.

Regarding uncertainty, we should further emphasize that our results come from a methodology which incorporates statistical parameterizations (geo-statistical model) over point data (REDES) and future climatic covariates (monthly precipitation, monthly maximum/minimum temperature, bioclimatic layers). Moreover, the results include high uncertainty due to the intrinsic climate model uncertainty. Consequently, the results should be regarded as an attempt to model future rainfall erosivity in Europe and identify differences in regional patterns.

### 3.5 Plausibility and comparison with local and regional studies

In this study, we showed that rainfall erosivity may on average increase by 18% in the European Union and Switzerland (zones which have generally similar characteristics to the ones in U.S.A) by 2050. For the U.S.A. Nearing et al. (2004) estimated a similar average increase of rainfall erosivity of around 17% in 2050. These matching results are due to very similar rainfall characteristics between the USA and Europe. Nonetheless, these changes are geographically variable.

Our results indicate that particularly changes of rainfall occurring during the warmest period of the year (April-September) would have high effects and increase rainfall erosivity. Diodato and Bellocchi (2010), identified the precipitation of autumn months as the major factor for their R-factor projections in the Mediterranean basin based only on monthly rainfall data. Highest erosivity values during summer and early autumn were also observed for major parts of the European Union (Panagos et al., 2016b) and Switzerland (Meusburger et al., 2012).

Contrasting trends of future rainfall erosivity have been identified for the Mediterranean basin (Fig. 5) which has complex geographical characteristics. According to Lionello et al. (2006), the complex morphology in the Mediterranean basin with distinct basins and gulfs and many sharp orographic features influences the sea and atmospheric circulation and lead to great spatial variability for precipitation.

According to the future climatic projections, mean annual precipitation would potentially increase in large parts of Central and Northern Europe by up to about 25% and decrease in Southern Europe (Kriegsmann et al., 2014). The heavy summer precipitation events, defined as events exceeding the intensity at the 95th percentile of daily precipitation, are modelled to decrease by about 25% in parts of Iberian Peninsula and Southern France accompanied by regional increases in parts of Spain and Portugal. Further, the heavy precipitation events in winter are modelled to increase by up to 25% in Central and Eastern Europe (Kriegsmann et al., 2014). Trends of the last 35 years already showed an increase (0.5 storm events per year) of the high-intensity storm events in lowland regions of Germany (Mueller and Pfister, 2011).

Christensen et al. (2015), focused on climate change in Europe and concluded that the change in very high precipitation extremes may have higher impact than the global temperature change. They also identified that higher scatter will take place in The British Isles and Middle Europe and lower scatter in the Mediterranean and Iberian Peninsula.

The predicted R-factor patterns mainly depend on the spatial patterns of the projected climatic covariates of the HadGEM2 model. The predicted rainfall erosivity increase in Northern and Central Europe is connected to the climate simulation model used in this study. Van Haren et al. (2013) predict an increase of both annual cumulated precipitation (especially in Northern and North-Eastern Europe) and frequency and intensity of extreme rainfall events, including the summer rain-shower and thunderstorms that can remarkably affect rainfall erosivity and subsequently soil erosion. On the other hand, the GCM used in this study still predicts the increase of extreme events in Southern Europe, but the annual cumulated rainfall is projected to significantly decrease there, in particular in the summer months, which are influencing the rainfall erosivity more than the winter months. We shall also highlight that the patterns described here are shared by most of the climate change scenario models commonly run in climate prediction experiment (for a detailed list see IPCC, 2013, Rajczak et al., 2013).

Even though the projections of rainfall erosivity are very plausible and congruent with other climate change studies, they may vary considerable depending on the choice of the scenario (e.g. within the RCP4.5 an increase of temperature in the range 0.9–2.0 Celsius degrees till 2046–2065 and compared to other ones). Moreover, the erosivity predictions also include uncertainties originating from the downscaling of GCMs. Besides the climate model uncertainty, the rainfall erosivity predictions embeds the natural variability of climate systems (Harris et al., 2014). Moreover, the rainfall projections have larger degrees of uncertainty compared to temperature projections because of a higher number of physical models involved and the generally higher variability of rainfall in space (Harris et al., 2014).

The projected erosivity dataset is not challenging any local (or regional) erosivity map which has been developed by using a different methodology or involved local data of better quality. Our erosivity projections were compared with the three regional studies modelling long-term R-factor measurements (i.e., Ukkel in Belgium, western Germany and Czech Republic). We observed good agreement in both trends and comparable magnitudes. In Belgium, Verstraeten et al. (2006) calculated 31% increase of erosivity during 20th Century compared to 40% increase that we project in 2050. In western Germany, Fiener et al. (2013) observed an R-factor increase of 21% per decade during the period 1973–2007 (overall about 70%). Here, we project for the next 40 years a trend consistent with the local long-term observations (+67%). Regarding the last study, Hanel et al. (2015) estimated in Czech Republic an increase of R-factor by 11% per decade (1960–1990) which is also in good agreement with our projection (+68% for the next 40 years).

Along with the quantitative comparison of our erosivity projections with local studies, we performed a further qualitative comparison of our results with regional studies which have modelled trends of future erosivity. Our results were compared well with local studies in Sicily and Calabria (Italy), Spain and North Ireland while the results were different in South Portugal. Similar to our results, Grauso et al. (2010) expect the higher values in the Catania plain and eastern slope of Iblei mountains while the lowest values are projected in south-east of Palermo. In Sicily, D'Asaro et al. (2007) are not expecting an increase of rainfall erosivity in the future. In Calabria, Capra et al. (2016) projected a decrease of R-factor, similar to our results (Fig. 5). In Ebro catchment (Spain), Angulo-Martínez and Beguería (2012) reported a decrease of very intense rainfall events but an increased frequency of moderate and low events which is close to our future projections (Fig. 5). Similar to our results, Mullan (2013) projected an increase of erosivity in western part of North Ireland (Corrard, Loughmuck) and a decrease of erosivity in eastern part (Dunadry, Hillsborough, Ballywalter). Contrary to our projections, Nunes et al. (2016) show a decrease of erosivity in Portugal (1950–2008) but an increase in precipitation concentration. In line to our study, Groisman et al. (2005) simulated an increase of heavy precipitations in Scandinavia and Northern Europe.

## 4 Conclusions and outlook

We modelled the rainfall erosivity in 2050 based on a moderate climate change scenario (HadGEM RCP 4.5) and using as main data sources the REDES based European R-factors and as covariates the WorldClim climatic datasets. Although the rainfall erosivity projections are based on many uncertainties, this pan-European spatial estimation highlights the areas where rainfall erosivity is projected to undergo substantial changes. The prediction of future erosivity in EU can contribute in policies related to soil/land and water sustainable management.

The overall increase of rainfall erosivity in Europe by 18% until 2050 are in line with projected increases of 17% for the U.S.A. The predicted R-factor dataset can be used for applying climate change scenarios in soil erosion models. The predicted mean increase in R-factor is expected also to increase the threat of soil erosion in Europe. However, climate change might substantially affect land cover and land use, which might counterbalance or enhance some erosional trends. In order to predict soil erosion trends in the future these feedbacks between rainfall erosivity and land use/land cover need to be considered. The most prominent increases of R-factors are predicted for North-Central Europe, the English Channel, The Netherlands and Northern France. On the contrary, parts of the Mediterranean basin show a decrease of rainfall erosivity.

The Gaussian Process Regression model applied showed a relatively good performance (R^2^ = 0.635, Relative error = 0.56) based on most of the monthly precipitation covariates of the WorldClim dataset. Despite this study significant contribution towards better understanding of future rainfall erosivity potential in Europe, the results should be in any case handled with care, as it should be commonly done with results derived from CCM and RCM models applied to future scenarios. Future research in climate change modelling will hopefully reduce the intrinsic climate model uncertainty and provide data on better spatial and temporal predictions of rainfall intensity trends. The projected rainfall erosivity (GeoTIFF format) at ∼ 1 km resolution will be available for free download in the European Soil Data Centre (ESDAC): http://esdac.jrc.ec.europa.eu/.

## Conflict of interest

The authors confirm and sign that there is no conflict of interest with networks, organisations and data centres referred to in this paper.
